# Oncogenic mutations within the β3‐αC loop of *EGFR*/*ERBB2*/*BRAF*/*MAP2K1* predict response to therapies

**DOI:** 10.1002/mgg3.1395

**Published:** 2020-08-05

**Authors:** Biao Zhang, Yongsheng Chen, Pingping Dai, Haoda Yu, Jianhui Ma, Chen Chen, Yan Zhang, Yanfang Guan, Rongrong Chen, Tao Liu, Jiayin Wang, Ling Yang, Xin Yi, Xuefeng Xia, Haitao Ma

**Affiliations:** ^1^ Department of Thoracic Surgery The First Affiliated Hospital of Soochow University Suzhou China; ^2^ Geneplus‐Beijing Beijing China; ^3^ Department of Computer Science and Technology School of Electronic and Information Engineering Xi'an Jiaotong University Xi'an China; ^4^ Department of Respiratory Medicine Wuxi People’s Hospital Wuxi China

**Keywords:** BRAF, EGFR, ERBB2, MAP2K1, Oncogenic mutations, β3‐αC loop

## Abstract

**Background:**

β3‐αC loop is a highly conserved structural domain across oncogene families, which is a switch for kinase activity. There have been numerous researches on mutations within β3‐αC loop in *EGFR*, but relatively less in *ERBB2*, *BRAF*, and *MAP2K1*. In addition, previous studies mainly focus on β3‐αC deletion in *EGFR*, which is the most common type affecting kinase activity and driving lung cancer. Other mutation types are not well studied.

**Methods:**

Here we analyzed the profile of β3‐αC loop mutations in a total of 10,000 tumor biopsy and/or ctDNA patient samples using hybridization capture‐based next‐generation sequencing.

**Results:**

We identified 1616 mutations within β3‐αC loop in this cohort. Most mutations were located in *EGFR*, with less percentage in *ERBB2*, *BRAF*, and *MAP2K1*. *EGFR* β3‐αC deletions occurred at a high percentage of 96.7% and were all drug‐relevant. We also detected rare *EGFR* β3‐αC insertions and point mutations, most of which were related to EGFR TKIs resistance. *ERBB2* β3‐αC deletions were only found in breast cancers and sensitive to EGFR/ERBB2 inhibitor. Moreover, *BRAF* and *MAP2K1* mutations within β3‐αC loop also demonstrated drugs relevance.

**Conclusion:**

Our study showed that oncogenic mutations within the β3‐αC loop of *ERBB2*, *MAP2K1*, and *BRAF* are analogous to that of *EGFR*, which have profound effect on drug response. Understanding the mutation profile within the β3‐αC loop is critical for targeted therapies.

## Graphical abstract

Oncogenic alterations within the kinase domain β3‐αC loop, which is highly conserved across oncogene families, are very common in human cancers. Therefore, understanding the spectrum of β3‐αC loop mutations within these genes is critical for target therapies. Motivated by this, we analyzed tumor biopsy and/or ctDNA samples of a large cohort of patients (10,000) using hybridization capture‐based next‐generation sequencing and summarized different alterations in this region and the possible response to target therapy. Our findings provided a comprehensive instruction for target therapies for cancer patients.

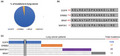

## 1 INTRODUCTION

Protein kinases usually have a structurally conserved domain and the way that the structural elements tend to fold determines the activation status of the kinases (Scheeff & Bourne, 2005). The structure will change in response to upstream signaling and thus lead to activation or inactivation of the kinases, which then regulates different biological activities. Mutations in this region or mutations that lead to the structure change will affect kinase activity and the downstream signaling. In one study, they analyzed somatic mutations across the kinome. They mapped significantly mutated positions (SMPs) of protein kinases and identified 23 SMPs. These SMPs are located at certain structural regions, such as activation loop (A‐loop), nucleotide binding P‐loop, the αC‐helix, the N‐ or C‐terminal to the αC‐helix (the β3‐αC loop and the αC‐β4 loop, respectively), and the catalytic loop (C‐loop; Kumar & Bose, 2017). β3‐αC loop in kinase domain is highly conserved across oncogene families. Deletions in the β3‐αC loop of *HER2* and *BRAF* have been shown to resemble *EGFR* exon 19 deletions (Foster et al., 2016). Exon 19 deletion within *EGFR* β3‐αC loop affecting kinase activity and driving lung cancer has been described (Chung et al., 2012).

Since kinase signaling pathways are involved in regulating multiple cellular processes, including proliferation, survival, motility, differentiation, and metabolism, activation of these signaling is frequently found in all kinds of tumors (Gross, Rahal, Stransky, Lengauer, & Hoeflich, 2015; Hanahan & Weinberg, 2011). Kinase activation can be caused by different mechanisms, such as point mutations, in‐frame deletions or insertions, and gene fusions (Foster et al., 2016).

There are a variety of anticancer drugs designed for targeting oncogenically activated kinases. The efficacy of some drugs could be affected by structure changes in the kinase domain. For example, drugs that target kinase activation in *BRAF*‐mutant cancers depend on structural variations in the BRAF kinase domain. *BRAF* exon 12 deletions within the β3‐αC loop, which is required for kinase activation, make the tumors resistant to “first‐generation” *BRAF*‐V600E inhibitors (Chakraborty et al., 2016). Recently, two new activating mutations of *BRAF* involving the β3‐αC loop in anaplastic pleomorphic xanthoastrocytoma were identified and patients with these mutations were predicted to be resistant to kinase inhibitors (Pratt et al., 2018). On the other hand, *BRAF* V600E mutation is often sensitive to small molecular kinase inhibitors (Cantwell‐Dorris, O'Leary, & Sheils, 2011; Holderfield, Deuker, McCormick, & McMahon, 2014).

Although β3‐αC loop widely exists in kinds of oncogenes, researches on mutations within this structure mainly focus on *EGFR* exon 19 deletions. Other mutation types and mutations in other oncogenes accommodating β3‐αC loop were not well understood. Here we analyze the profile of β3‐αC loop mutations within important oncogenes, *EGFR*, *ERBB2*, *BRAF*, and *MAP2K1*, and the potential implications on sensitivity to therapeutic drugs in Chinese patients.

## 2 MATERIALS AND METHODS

### 2.1 Patients and sample collection

Between October 2015 and July 2018, 10,000 patients with advanced cancer from China received next‐generation sequencing (NGS) testing in Geneplus‐Beijing Institute. For each patient, signed consent form was obtained before starting the test. Tissue including fresh, formalin‐fixed, paraffin‐embedded samples, or plasma samples was obtained. Tumor specimens were reviewed by qualified pathologists to confirm the diagnosis of tumor types and ensure > 20% tumor content. At the same time, peripheral blood was collected using EDTA Vacutainer tubes (BD Diagnostics) as a source of matched normal (germline) sample. As for plasma testing, 10‐ml peripheral blood was collected from each patient in cell‐free DNA BCT tubes (Streck) and processed within 72 hr of sample collection.

### 2.2 Sample processing and DNA extraction

Extraction of DNA was performed as previously described (Nong et al., 2018). Genomic DNA was extracted from FFPE samples using Maxwell^®^ RSC DNA FFPE Kit (Promega). Plasma was separated by centrifugation at 1,600 *g* for 10 min, transferred to new microcentrifuge tubes, and centrifuged at 16,000 *g* for 10 min to remove remaining cell debris. Peripheral blood lymphocytes (PBLs) from the initial spin were used for isolation of germline genomic DNA. PBL DNA was extracted using the DNeasy Blood & Tissue Kit (Qiagen). Cell‐free circulating DNA was isolated from plasma using QIAamp Circulating Nucleic Acid Kit (Qiagen). DNA concentration was measured using a Qubit fluorometer and the Qubit dsDNA HS (High Sensitivity) Assay Kit (Invitrogen, Carlsbad). With that, the size distribution of plasma DNA was assessed using an Agilent 2100 BioAnalyzer and the DNA HS kit (Agilent Technologies).

### 2.3 Sequencing library construction and target enrichment

Before library construction, genomic DNA extracted from PBL or FFPE specimen was sheared to 300 bp fragments with a Covaris S2 ultrasonicator (Covaris). KAPA Library Preparation Kit (Kapa Biosystems) was adopted to prepare indexed Illumina NGS libraries from PBL DNA, tumor DNA, and plasma DNA. Target enrichment was performed with a custom SeqCap EZ Library (Roche NimbleGen). The capture probe designed based on cancer genomic regions of 59 genes or 1021 genes was used in this study to explore the comprehensive genetic properties of advanced solid tumors. Capture hybridization was carried out according to the manufacturer's protocol. Following hybrid selection, the captured DNA fragments were amplified and then pooled to generate several multiplex libraries.

### 2.4 NGS Sequencing and data analysis

Sequencing was carried out using Illumina 2 × 75 bp paired‐end sequencing on Illumina HiSeq 3000 instruments, using TruSeq PE Cluster Generation Kit v3 and the TruSeq SBS Kit v3 (Illumina) according to the manufacturers’ recommendations. After removal of terminal adaptor sequences and low‐quality data, reads were mapped to the reference human genome (hg19) and aligned using BWA (0.7.12‐r1039; Li & Durbin, 2009).

GATK (https://www.broadinstitute.org/gatk/, The Genome Analysis Toolkit), MuTect2 (3.4‐46‐gbc02625; Cibulskis et al., 2013), and in‐house algorithm NChot (Yang et al., 2017) were employed to call somatic small insertions and deletions (indel), and single nucleotide variants (SNV) by filtering PBL sequencing data. Contra (2.0.8) was used to detect copy number variations (Li et al., 2012). For structure variations (SV), an in‐house algorithm NCSV was used to identify split‐read and discordant read‐pair to identify SVs (Yang et al., 2020).

For the sequence variant nomenclature, we followed the Human Genome Variation Society (HGVS) recommendations and used the transcript references as below: *EGFR* NM_005228.3, *ERBB2* NM_004448.2, *BRAF* NM_004333.4, *MAP2K1* NM_002755.3.

### 2.5 Actionable mutation assessment

Sequence mutations were annotated according to in‐house database, a curated knowledge base of the oncogenic effects and treatment implications of somatic mutations. According to the level of evidence that the mutation is a predictive biomarker of drug response, mutations were classified in a tumor type‐specific manner.

## 3 RESULTS

### 3.1 Key genetic alterations identified in patients

**Figure 1:**
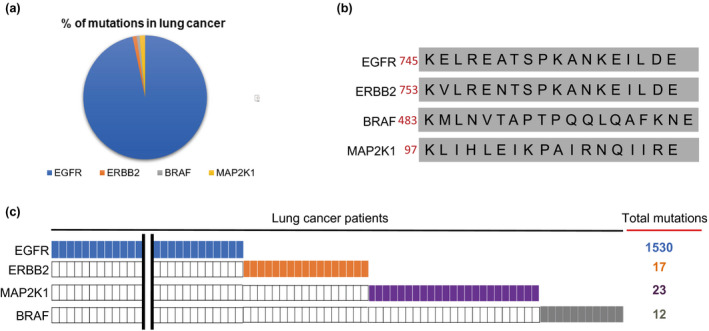
Distribution of key mutations within β3‐αC loop of protein kinases *EGFR*, *ERBB2*, *BRAF*, and *MAP2K1*. (a) Percentage of each mutation in lung cancer samples. (b) Amino acid sequence of β3‐αC loop of protein kinases *EGFR*, *ERBB2*, *BRAF*, and *MAP2K1*. (c) Mutations in β3‐αC loop of protein kinases *EGFR*, *ERBB2*, *BRAF*, and *MAP2K1* in lung cancer are mutually exclusive. The transcript references are as below: *EGFR* NM_005228.3, *ERBB2* NM_004448.2, *BRAF* NM_004333.4, *MAP2K1* NM_002755.3

Total of 10,000 patients diagnosed with advanced cancer between October 2015 and July 2018 were included in this study, with 65.5% of all patients were lung cancer, 7.3% were breast cancer, 1.4% were pancreatic cancer, 7.6% were colorectal, and 18.1% were other cancer types. All patients received NGS testing. We identified 1616 mutations within β3‐αC loop of protein kinases EGFR, ERBB2, BRAF, and MAP2K1 in the cohort. Of these mutations, 97.8% (1583/1616) were found in lung cancer, with 96.71% (1530/1583) of the mutations in lung cancer were located in *EGFR*, 1.1% (17/1583) located in *ERBB2*, 0.7% (12/1583) located in *BRAF*, and 1.4% (23/1583) in *MAP2K1* (Table 1 and Figure 1a). Of all mutations identified here, 1.2% (20/1617), 0.25% (4/1617), 0.19% (3/1617), and 0.49% (8/1617) were found in breast cancer, colorectal cancer, pancreatic cancer, and others, respectively. Hundred percent of the mutations found in breast cancer were located in *ERBB2* and similarly, *BRAF* accounts for all mutations in colorectal cancer and *MAP2K1* in pancreatic cancers. The kinase domain β3‐αC loop is highly conserved across oncogenes and also resides in ERBB2, BRAF, and MAP2K1 (Figure 1b). Interestingly, we found that mutations in this region of *EGFR*, *BRAF*, *ERBB2*, and *MAP2K1* were mutually exclusive (Figure 1c), which is consistent with a previous report (Arcila et al., 2015) that suggested one driver mutation is sufficient cancer initiation or development.

**Table 1:** Distribution of identified mutations in different cancer types

	Total mutation	Genes	Lung cancer	Breast cancer	Colorectal cancer	Pancreatic cancer	Other cancers
	1530	EGFR	1,530 (96.7%)	—	—	—	—
	38	ERBB2	17 (1.07%)	20 (100%)	—	—	1 (14%)
	18	BRAF	12 (0.70%)	—	—	3 (100%)	3 (43%)
	30	MAP2K1	23 (1.45%)	—	4 (100%)	—	3 (43%)
Total	1616	—	1583 (97.8%)	20 (1.2%)	4 (0.25%)	3 (0.19%)	7 (0.43%)

Note

Number in brackets indicates the percentage of certain type of cancer that harbors the corresponding gene mutation. The transcript references are as below: *EGFR* NM_005228.3, *ERBB2* NM_004448.2, *BRAF* NM_004333.4, *MAP2K1* NM_002755.3.

### 3.2 Characteristics of *EGFR* mutations and clinical indication

All *EGFR* β3‐αC alterations were found in lung cancer patients and *EGFR* β3‐αC deletions were present in a very high frequency of 96.7% (1479/1530), with the most frequent deletion of amino acids glutamic acid‐leucine‐arginine‐glutamic acid‐alanine (also referred to as ΔELREA), followed by lower frequencies of ∆LREATS, P753S, and ∆LREAT. We also found 12 patients harboring rare *EGFR* β3‐αC insertion (I740_K745dup, also referred to as K745_E746insIPVAIK), which was reported as tyrosine kinase inhibitor (TKI)‐sensitive mutation (Kobayashi & Mitsudomi, 2016). Although the therapeutic response of patients with *EGFR* β3‐αC deletions to different EGFR TKIs was slightly different, all the patients with the deletions were sensitive to EGFR TKI treatment. This is consistent with previous study that patients with LRE deletions were associated with better response to TKIs than those with non‐LRE deletions in exon 19 (Chung et al., 2012). We were also able to identify 38 (24.8%, 38/1530) missense mutations within β3‐αC region and among these, 34% (13/38), that is p.L747P and 3 p.L747S (Table 2), showed influence on kinase activities and were found to be resistant to EGFR TKI treatment according to previous studies (Liu, Wu, Zhong, Hui, & Fang, 2013; Wang, Ning, Li, & Huang, 2016; Wu et al., 2011; Yamaguchi et al., 2014; Yamaguchi, Kugawa, Tateno, Kokubu, & Fukuchi, 2012; Yu et al., 2015).

**Table 2:** Major alterations in β3‐αC region of EGFR, ERBB2, BRAF, and MAP2K1 genes in lung cancer

	EGFR_mis	EGFR β3‐αC delins	ERBB2	MAP2K1	BRAF mutations
	p.L747P (10)	p.E746_A750del (978)	p.L755P (5)	p.E102_I103del (15)	p.N486_P490del (2)
	p.V742I (6)	p.L747_P753delinsS (131)	p.D769Y (2)	p.P105_A106del (4)	p.K483E (3)
	p.V742L (5)	p.L747_T751del (79)	p.K753E (2)	p.Y134F (1)	p.D479N (2)
	p.D761Y (4)	p.L747_A750delinsP (66)	p.I767M (1)	p.A106T (1)	p.L485F (1)
	p.L747S (3)	p.E746_T751delinsA (31)	p.D769H (1)	p.V127M (1)	p.E501K (1)
	p.I744M (2)	p.E746_S752delinsV (28)	p.I740M (1)	p.I99V (1)	p.K483Q (1)
	p.I759M (2)	p.L747_S752del (22)	p.L607Q (1)		p.Q496* (1)
	p.K757M (2)	p.L747_T751delinsP (16)	p.L755V (1)		p.N486_A489delinsK (1)
	p.E734D (1)	p.L747_E749del (12)	p.S760del (1)		
	p.I759N (1)	p.I740_K745dup (12)	p.I767F (1)		
	p.K757L (1)	Others together (117)	p.L755S (1)		
	p.L747C (1)				
DR	34% (13/38)^a^	100%^a^	59% (10/17)^‡^	70% (16/23)^§^	50% (6/12)^¶^
Total	38	1,492	17	23	12

Note

DR, percentage of drug‐relevant mutations; Red color indicates the drug‐relevant mutations. The transcript references are as below: *EGFR* NM_005228.3, *ERBB2* NM_004448.2, *BRAF* NM_004333.4, *MAP2K1* NM_002755.3.

^a^ Sensitive to EGFR TKI.

^b^ Related to the efficacy of HER2 target therapy.

^c^ Related to MEK target therapy.

^d^ Related to BRAF/MEK target therapy.

### 3.3 Characteristics of *ERBB2*, *BRAF*, and *MAP2K1* mutations and clinical indication

*ERBB2* β3‐αC deletions (ΔLRENT) were only detected in breast cancers, which were sensitive to EGFR/ERBB2 inhibitor neratinib and resistant to lapatinib. In lung cancer, most of the *ERBB2* β3‐αC mutations are point mutations and the percentage of drug‐relevant mutations was 59% (10/17; Table 2, red). Sixty‐one percent (11/18) of *BRAF* mutations were found in lung cancers and 16.7% (3/18) in pancreatic cancers. About 45.5% (5/11) of lung cancer patients with *BRAF* β3‐αC loop mutations demonstrated drug relevance. We detected 23 (23/30 = 76.7%) *MAP2K1* mutations in lung cancer patients and four (4/30 = 13.3%) in colorectal cancer patients. In contrast to *ERBB2*, most of *MAP2K1* mutations found in lung cancer are β3‐αC deletions. Seventy percent (15/23) of the mutations in this region were drug‐relevant.

## 4 DISCUSSION

In this study, we analyzed a large number of 10,000 patients with advanced cancers for oncogenic mutations in *EGFR*, *ERBB2*, *BRAF*, and *MAP2K1*, especially for those located within the kinase domain β3‐αC loop. In addition, we summarized the effect of these mutations on the sensitivity to target therapy, that is TKIs. These data not only refined our understanding of the mutation profile within β3‐αC loop of these kinase domain but also provided a comprehensive instruction for target therapies.

Most kinase domain β3‐αC loop deletions/insertions or point mutations are activating mutations as shown in a previous study (Foster et al., 2016). We found an interesting phenomenon that mutations in this region of *EGFR*, *BRAF*, *ERBB2*, and *MAP2K1* were mutually exclusive, indicating changes in this region of these kinases function similarly to drive tumorigenesis. This finding supports the opinion that single driver mutation is required for oncogene addiction in lung tumors (Gazdar, Shigematsu, Herz, & Minna, 2004). *EGFR* is one of the most frequently altered genes in lung cancer, especially in Asian adenocarcinoma subgroup (Dearden, Stevens, Wu, & Blowers, 2013). Most EGFR gene mutations can be targeted for therapy and thus is considered the most common “actionable” mutation in lung cancer patients (Castellanos, Feld, & Horn, 2017). In our study, most of the kinase β3‐αC loop alterations found in lung cancer were from *EGFR* with all β3‐αC indels, and 34% of point mutations in this region were drug‐relevant alterations. About 59% *ERBB2* mutations, 65% *MAP2K1* mutations, and 50% *BRAF* mutations in this region were drug‐relevant, confirming the importance of β3‐αC loop for kinase functions.

Genetic alterations in MAPK/ERK pathway are very common in non‐small cell lung cancer (NSCLC), especially mutations found in the upstream members, *EGFR* and *KRAS*, which account for 70% or more of all known driver mutations (Ladanyi & Pao, 2008; Riely et al., 2008). Alterations in *BRAF* and *MAP2K1* (*MEK1*) are less common, with *BRAF* mutations well studied and *MAP2K1* mutation remaining relatively undefined with a close association with smoking (Arcila et al., 2015; Marks et al., 2008; Paik et al., 2011). In our study, we found similar genetic alterations in kinase β3‐αC loop and that the majority of them are localized in EGFR gene. Human epidermal growth factor receptor 2 (*HER2*; *ERBB2*) is tyrosine kinase receptor and the mutation or amplification of which have been linked with human cancers, with lung cancer and breast cancer the most common (Connell & Doherty, 2017). We found about 52.6% (20/38) of *ERBB2* mutations were from breast cancer patients and 44.7% (17/38) were from lung cancer patients, with 59% of all mutations in β3‐αC loop were druggable.

In conclusion, based on a large cohort of cancer patients, our study analyzed mutations in β3‐αC loop in kinase domain of *EGFR*, *ERBB2*, *MAP2K1*, and *BRAF*, and summarized the drug‐relevant mutations of each gene. These results highlighted the significance of β3‐αC loop in kinase domain and generated valuable mutation profiles of this region, thus providing insight into the target therapies that rely on these mutations.

## CONFLICT OF INTEREST

The authors declare that there is no conflict of interest regarding the publication of this paper.

## AUTHOR CONTRIBUTIONS

Collection of clinical data: Haoda Yu, Jiayin Wang, Ling Yang, and Xin Yi; Data analyzed and interpreted: Yongsheng Chen, Yan Zhang, Yanfang Guan, Rongrong Chen, and Tao Liu; Writing and review of original draft of the manuscript: Biao Zhang, Jianhui Ma, Pingping Dai, ChenChen, Xuefeng Xia, and Haitao Ma.

## Data Availability

The data that support the findings of this study are available from the corresponding author upon reasonable request.

## References

[mgg31395-bib-0001] Arcila, M. E. , Drilon, A. , Sylvester, B. E. , Lovly, C. M. , Borsu, L. , Reva, B. , … Ladanyi, M. (2015). MAP2K1 (MEK1) mutations define a distinct subset of lung adenocarcinoma associated with smoking. Clinical Cancer Research, 21(8), 1935–1943. 10.1158/1078-0432.CCR-14-2124 25351745 PMC4401580

[mgg31395-bib-0002] Cantwell‐Dorris, E. R. , O'Leary, J. J. , & Sheils, O. M. (2011). BRAFV600E: Implications for carcinogenesis and molecular therapy. Molecular Cancer Therapeutics, 10(3), 385–394. 10.1158/1535-7163.MCT-10-0799 21388974

[mgg31395-bib-0003] Castellanos, E. , Feld, E. , & Horn, L. (2017). Driven by mutations: the predictive value of mutation subtype in egfr‐mutated non‐small cell lung cancer. Journal of Thoracic Oncology, 12(4), 612–623. 10.1016/j.jtho.2016.12.014 28017789

[mgg31395-bib-0004] Chakraborty, R. , Burke, T. M. , Hampton, O. A. , Zinn, D. J. , Lim, K. P. H. , Abhyankar, H. , … Allen, C. E. (2016). Alternative genetic mechanisms of BRAF activation in Langerhans cell histiocytosis. Blood, 128(21), 2533–2537. 10.1182/blood-2016-08-733790 27729324 PMC5123197

[mgg31395-bib-0005] Chung, K.‐P. , Wu, S.‐G. , Wu, J.‐Y. , Yang, J.‐C.‐H. , Yu, C.‐J. , Wei, P.‐F. , … Yang, P.‐C. (2012). Clinical outcomes in non‐small cell lung cancers harboring different exon 19 deletions in EGFR. Clinical Cancer Research, 18(12), 3470–3477. 10.1158/1078-0432.CCR-11-2353 22510346

[mgg31395-bib-0006] Cibulskis, K. , Lawrence, M. S. , Carter, S. L. , Sivachenko, A. , Jaffe, D. , Sougnez, C. , … Getz, G. (2013). Sensitive detection of somatic point mutations in impure and heterogeneous cancer samples. Nature Biotechnology, 31(3), 213–219. 10.1038/nbt.2514 PMC3833702 23396013

[mgg31395-bib-0007] Connell, C. M. , & Doherty, G. J. (2017). Activating HER2 mutations as emerging targets in multiple solid cancers. ESMO Open, 2(5), e000279 10.1136/esmoopen-2017-000279 29209536 PMC5708307

[mgg31395-bib-0008] Dearden, S. , Stevens, J. , Wu, Y. L. , & Blowers, D. (2013). Mutation incidence and coincidence in non small‐cell lung cancer: Meta‐analyses by ethnicity and histology (mutMap). Annals of Oncology, 24(9), 2371–2376. 10.1093/annonc/mdt205 23723294 PMC3755331

[mgg31395-bib-0009] Foster, S. A. , Whalen, D. M. , Özen, A. , Wongchenko, M. J. , Yin, J. P. , Yen, I. , … Malek, S. (2016). Activation mechanism of oncogenic deletion mutations in BRAF, EGFR, and HER2. Cancer Cell, 29(4), 477–493. 10.1016/j.ccell.2016.02.010 26996308

[mgg31395-bib-0010] Gazdar, A. F. , Shigematsu, H. , Herz, J. , & Minna, J. D. (2004). Mutations and addiction to EGFR: The Achilles 'heal' of lung cancers? Trends in Molecular Medicine, 10(10), 481–486. 10.1016/j.molmed.2004.08.008 15464447

[mgg31395-bib-0011] Gross, S. , Rahal, R. , Stransky, N. , Lengauer, C. , & Hoeflich, K. P. (2015). Targeting cancer with kinase inhibitors. Journal of Clinical Investigation, 125(5), 1780–1789. 10.1172/JCI76094 25932675 PMC4463189

[mgg31395-bib-0012] Hanahan, D. , & Weinberg, R. A. (2011). Hallmarks of cancer: The next generation. Cell, 144(5), 646–674. 10.1016/j.cell.2011.02.013 21376230

[mgg31395-bib-0013] Holderfield, M. , Deuker, M. M. , McCormick, F. , & McMahon, M. (2014). Targeting RAF kinases for cancer therapy: BRAF‐mutated melanoma and beyond. Nature Reviews Cancer, 14(7), 455–467. 10.1038/nrc3760 24957944 PMC4250230

[mgg31395-bib-0014] Kobayashi, Y. , & Mitsudomi, T. (2016). Not all epidermal growth factor receptor mutations in lung cancer are created equal: Perspectives for individualized treatment strategy. Cancer Science, 107(9), 1179–1186. 10.1111/cas.12996 27323238 PMC5021039

[mgg31395-bib-0015] Kumar, R. D. , & Bose, R. (2017). Analysis of somatic mutations across the kinome reveals loss‐of‐function mutations in multiple cancer types. Scientific Reports, 7(1), 6418 10.1038/s41598-017-06366-x 28743916 PMC5527104

[mgg31395-bib-0016] Ladanyi, M. , & Pao, W. (2008). Lung adenocarcinoma: Guiding EGFR‐targeted therapy and beyond. Modern Pathology, 21(Suppl 2), S16–22. 10.1038/modpathol.3801018 18437168

[mgg31395-bib-0017] Li, H. , & Durbin, R. (2009). Fast and accurate short read alignment with Burrows‐Wheeler transform. Bioinformatics, 25(14), 1754–1760. 10.1093/bioinformatics/btp324 19451168 PMC2705234

[mgg31395-bib-0018] Li, J. , Lupat, R. , Amarasinghe, K. C. , Thompson, E. R. , Doyle, M. A. , Ryland, G. L. , … Gorringe, K. L. (2012). CONTRA: Copy number analysis for targeted resequencing. Bioinformatics, 28(10), 1307–1313. 10.1093/bioinformatics/bts146 22474122 PMC3348560

[mgg31395-bib-0019] Liu, Y. , Wu, B. Q. , Zhong, H. H. , Hui, P. , & Fang, W. G. (2013). Screening for EGFR and KRAS mutations in non‐small cell lung carcinomas using DNA extraction by hydrothermal pressure coupled with PCR‐based direct sequencing. International Journal of Clinical and Experimental Pathology, 6(9), 1880–1889. 24040454 PMC3759496

[mgg31395-bib-0020] Marks, J. L. , Gong, Y. , Chitale, D. , Golas, B. , McLellan, M. D. , Kasai, Y. , … Pao, W. (2008). Novel MEK1 mutation identified by mutational analysis of epidermal growth factor receptor signaling pathway genes in lung adenocarcinoma. Cancer Research, 68(14), 5524–5528. 10.1158/0008-5472.CAN-08-0099 18632602 PMC2586155

[mgg31395-bib-0021] Nong, J. , Gong, Y. , Guan, Y. , Yi, X. , Yi, Y. , Chang, L. , … Wang, J. (2018). Circulating tumor DNA analysis depicts subclonal architecture and genomic evolution of small cell lung cancer. Nature Communications, 9(1), 3114 10.1038/s41467-018-05327-w PMC6079068 30082701

[mgg31395-bib-0022] Paik, P. K. , Arcila, M. E. , Fara, M. , Sima, C. S. , Miller, V. A. , Kris, M. G. , … Riely, G. J. (2011). Clinical characteristics of patients with lung adenocarcinomas harboring BRAF mutations. Journal of Clinical Oncology, 29(15), 2046–2051. 10.1200/JCO.2010.33.1280 21483012 PMC3107760

[mgg31395-bib-0023] Pratt, D. , Camelo‐Piragua, S. , McFadden, K. , Leung, D. , Mody, R. , Chinnaiyan, A. , … Venneti, S. (2018). BRAF activating mutations involving the beta3‐alphaC loop in V600E‐negative anaplastic pleomorphic xanthoastrocytoma. Acta Neuropathologica Communications, 6(1), 24 10.1186/s40478-018-0525-1 29544532 PMC5855983

[mgg31395-bib-0024] Riely, G. J. , Kris, M. G. , Rosenbaum, D. , Marks, J. , Li, A. , Chitale, D. A. , … Ladanyi, M. (2008). Frequency and distinctive spectrum of KRAS mutations in never smokers with lung adenocarcinoma. Clinical Cancer Research, 14(18), 5731–5734. 10.1158/1078-0432.CCR-08-0646 18794081 PMC2754127

[mgg31395-bib-0025] Scheeff, E. D. , & Bourne, P. E. (2005). Structural evolution of the protein kinase‐like superfamily. PLoS Computational Biology, 1(5), e49 10.1371/journal.pcbi.0010049 16244704 PMC1261164

[mgg31395-bib-0026] Wang, Y. T. , Ning, W. W. , Li, J. , & Huang, J. A. (2016). Exon 19 L747P mutation presented as a primary resistance to EGFR‐TKI: A case report. Journal of Thoracic Disease, 8(7), E542–546. 10.21037/jtd.2016.05.95 PMC4958842 27499993

[mgg31395-bib-0027] Wu, J. Y. , Yu, C. J. , Chang, Y. C. , Yang, C. H. , Shih, J. Y. , & Yang, P. C. (2011). Effectiveness of tyrosine kinase inhibitors on "uncommon" epidermal growth factor receptor mutations of unknown clinical significance in non‐small cell lung cancer. Clinical Cancer Research, 17(11), 3812–3821. 10.1158/1078-0432.Ccr-10-3408 21531810

[mgg31395-bib-0028] Yamaguchi, F. , Fukuchi, K. , Yamazaki, Y. , Takayasu, H. , Tazawa, S. , Tateno, H. , … Kokubu, F. (2014). Acquired resistance L747S mutation in an epidermal growth factor receptor‐tyrosine kinase inhibitor‐naïve patient: A report of three cases. Oncology Letters, 7(2), 357–360. 10.3892/ol.2013.1705 24396447 PMC3881940

[mgg31395-bib-0029] Yamaguchi, F. , Kugawa, S. , Tateno, H. , Kokubu, F. , & Fukuchi, K. (2012). Analysis of EGFR, KRAS and P53 mutations in lung cancer using cells in the curette lavage fluid obtained by bronchoscopy. Lung Cancer, 78(3), 201–206. 10.1016/j.lungcan.2012.08.014 23026641

[mgg31395-bib-0030] Yang, J. , Gong, Y. , Lam, V. K. , Shi, Y. , Guan, Y. , Zhang, Y. , … Yu, P. (2020). Deep sequencing of circulating tumor DNA detects molecular residual disease and predicts recurrence in gastric cancer. Cell Death & Disease, 11(5), 1–9. 10.1038/s41419-020-2531-z 32393783 PMC7214415

[mgg31395-bib-0031] Yang, X. , Chu, Y. , Zhang, R. , Han, Y. , Zhang, L. , Fu, Y. U. , … Li, J. (2017). Technical validation of a next‐generation sequencing assay for detecting clinically relevant levels of breast cancer‐related single‐nucleotide variants and copy number variants using simulated cell‐free DNA. The Journal of Molecular Diagnostics, 19(4), 525–536. 10.1016/j.jmoldx.2017.04.007 28502728

[mgg31395-bib-0032] Yu, G. , Xie, X. , Sun, D. , Geng, J. , Fu, F. , Zhang, L. , & Wang, H. (2015). EGFR mutation L747P led to gefitinib resistance and accelerated liver metastases in a Chinese patient with lung adenocarcinoma. International Journal of Clinical and Experimental Pathology, 8(7), 8603–8606. 26339441 PMC4555769

